# Comparative study of bladder augmentation using different biomimetic scaffolds: electrospun nanofiber vs. extracellular matrix scaffold with adipose-derived stem cells

**DOI:** 10.1097/JS9.0000000000003152

**Published:** 2025-08-08

**Authors:** Xuesheng Wang, Fan Zhang, Zhonghan Zhou, Qiang Fu, Limin Liao

**Affiliations:** a Department of Urology, Shandong Provincial Hospital Affiliated to Shandong First Medical University, Jinan, China; b Department of Urology, China Rehabilitation Research Center, School of Rehabilitation of Capital Medical University, Beijing, China; c University of Health and Rehabilitation Sciences, Qingdao, China; d China Rehabilitation Science Institute, Beijing, China; e Cheeloo College of Medicine, Shandong University, Jinan, China; f Key Laboratory of Urinary diseases, Shandong First Medical University, Jinan, China

**Keywords:** adipose-derived stem cells, bladder augmentation and reconstruction, nanofiber scaffolds, small intestinal submucosa, tissue engineering

## Abstract

**Background:**

Bladder augmentation with gastrointestinal segments is a widely used surgical procedure for neurogenic bladder, but it carries a risk of many side effects. However, there have been no systematic studies on which biomaterials are suitable for bladder augmentation and reconstruction. The aim of this study was to compare the safety and applicability of small intestinal submucosa (SIS), poly(l-lactic) acid (PLLA) nanofibrous scaffold, and PLLA/gelatin composite nanofibrous scaffold as a potential bladder wall substitute material in tissue-engineered bladder augmentation and reconstruction. The results provide a scientific basis for selecting appropriate materials in clinical applications.

**Materials and Methods:**

The microstructure, cytocompatibility, cell adhesion, and histocompatibility of scaffolds including SIS, PLLA nanofiber scaffold, and PLLA/Gelatin were observed. Furthermore, bladder augmentation rabbit models were constructed using scaffolds with and without adipose-derived stem cells (ASCs) implantation. Cystography and urodynamic examination were performed to evaluate the morphology and function of the reconstructed bladder. Histologic and immunofluorescence were used to assess the regeneration status of the reconstructed bladder.

**Results:**

Compared to SIS and PLLA/Gelatin scaffolds, the PLLA scaffold possessed appropriate mechanical properties, pore size and porosity, which could facilitate suturing, maintain bladder form, and promote the adhesion and proliferation of seeded ASCs. All animals survived in the experiment with no complications, and the structural integrity of the implantation site was demonstrated using cystography and urodynamics. Histological and immunohistochemical analyses indicated that the three kinds of scaffold could regenerate the bladder wall structure at 6 and 12 weeks. Bladder reconstructed with the ASCs-PLLA scaffold showed superior structural and functional properties, with no significant differences in the regenerated urothelium, smooth muscle, or vessels of the ASCs-PLLA and control groups.

**Conclusion:**

The ASCs-PLLA scaffold – composed of PLLA with favorable biological properties and ASCs with facilitating regeneration – presents a promising candidate as an ideal scaffold for bladder augmentation and reconstruction.

## Introduction

The primary function of the bladder is to provide an allantoic capsule and enable controlled urination. However, the bladder dysfunction resulting from dysplasia, cancer, or neurologic diseases can lower quality of life and be life-threatening. Bladder reconstruction was considered a viable option for management of refractory lower urinary tract dysfunction resulting from progressive declines in bladder function^[1]^. Currently, bladder reconstruction with autogenous or exogenous gastrointestinal segments is regarded as the standard surgical procedure for those diseases^[2]^. It was worth noting that postoperative complications such as metabolic disturbance, infection, urolithiasis, and even malignancy^[3]^ had plagued most patients. To avoid these complications, innovative tissue engineering has become an alternative to traditional surgical treatment. With the development of tissue engineering technology, diverse natural or synthetic materials with distinct properties, with or without seeded cells and/or biologic factors, were used for bladder tissue regeneration^[4]^.

Bladder substitutes derived from porcine small intestinal submucosa (SIS) or bladder acellular matrix (BAM), are typically made of extracellular matrix (ECM) that removes cells and other immunogenic components^[5]^. These substitutes are rich in many components, including collagen, proteoglycans, and functional growth factors, and facilitate the proliferation and differentiation of cells^[6,7]^. However, clinical applications of those materials may lead to calculi formation due to poor degradation rate^[8,9]^, bladder rupture caused by low mechanical strength^[8,10]^, and immunologic problems leading to fibrosis^[11,12]^. In addition, the therapeutic effect of native-derived substitutes may require more effort to guarantee the homogeneity of products before full clinical application. On the other hand, absorbable synthetic material is an alternative due to its malleable properties, stable repeatability, and good processability, which can be easily tailored into required shapes and sizes. Poly(l-lactic) acid (PLLA) has been widely used in different areas of biomedicine by virtue of its advantages in terms of its mechanical, thermoplastic, biocompatible, and biodegradable properties^[13]^. Previous studies have confirmed that PLLA is a promising material employed in bladder tissue engineering^[13,14]^. In addition, a composite and biomimetic, nonwoven fiber scaffold composed of PLLA and gelatin also has proven to repair dura tissue defects with optimal clinical effects^[5,15]^.

Stem cells have high self-renewal and multidirectional differentiation capabilities and an essential role in tissue regeneration^[16]^. In tissue engineering and regenerative medicine, autologous adipose-derived stem cells (ASCs) are now relatively abundant, are readily harvested, and offer multipotential differentiation.

Currently, biodegradable materials derived from natural or synthetic matrix, generally defined as “bioprosthetic meshes,” are widely used in clinical surgical practice^[17,18]^. However, there have been no systematic studies or reviews to conducted to determine which biomaterials work best on tissue-engineered bladder augmentation and reconstruction. Hence, the purpose of our study was to investigate the safety and applicability of SIS, PLLA and PLLA/Gelatin biomimetic materials, which are typical representatives of natural acellular matrix, synthetic polymer materials and composite materials, in tissue-engineered bladder augmentation. The findings from our study can provide scientific evidence for selecting an appropriate material for clinical application and *in situ* three-dimensional (3D) bioprinting of bladder tissue. The safety and effectiveness were systematically evaluated via *in vitro* and *in vivo* experiments. Biological properties of these scaffolds including cytotoxicity, cell adhesion and distribution, proliferation, and stemness maintenance, were compared in detail in our study. In addition, we also investigated the performance of reconstructed bladder using urodynamics and compared the histologic differences of tissue repair sites in the animal model. Furthermore, we evaluated the efficacy of scaffolds with or without seed cells to guide tissue regeneration. Our research has been reported in line with the TITAN criteria^[19]^.

## Materials and methods

### Materials

Both the PLLA nanofiber scaffold (ReDura™) and PLLA/gelatin nanofiber scaffold (Neodura^TM^), manufactured by electrospinning technology, were provided by Medprin Biotech GmbH (Frankfurt am Main, Germany). The fibrous structure of these materials is similar to the ECM of human organ tissues^[5,15]^. The substitute used in this study was SIS from Cook Biotech (West Lafayette, IN, USA), which is fabricated with four layers of porous fibrillar collagen type I.

**HIGHLIGHTS**

- Bladder reconstruction with autogenous or exogenous gastrointestinal segments is regarded as the standard surgical procedure for those diseases. It was worth noting that postoperative complications such as metabolic disturbance, infection, urolithiasis, and even malignancy had plagued most patients.
- Biodegradable materials derived from natural or synthetic matrix, generally defined as “bioprosthetic meshes,” are widely used in clinical practice. However, it is rarely used in studies of bladder function reconstruction
- However, there have been no systematic studies or reviews to conducted to determine which biomaterials work best on tissue-engineered bladder augmentation and reconstruction.
- The findings from our study can provide the scientific evidence for selecting an appropriate material for clinical application and *in situ* three-dimensional (3D) bioprinting of bladder tissue.

### Cell cultures

Rabbit ASCs (Procell Life Science & Technology Co. Ltd., China) were cultured in rabbit ASCs culture medium (Cyagen Biosciences, China) with 10% (volume fraction) fetal bovine serum (FBS, Gibco). The culture medium containing cells was incubated in a humidified chamber (5% carbon dioxide) at 37°C, with medium changes every 2 or 3 days. ASCs were harvested when cells reached 80–85% confluence, and passages three and four were used for the experiments. In addition, rabbit bladder epithelial cells (Procell Life Science & Technology Co. Ltd., China) were cultured in F12K medium (Servicebio, China) with 10% FBS.

### Scaffold treatment

Prior to constructing the complex of ASCs-scaffold, the SIS, PLLA and PLLA/Gelatin scaffold materials were immersed in phosphate-buffered saline (PBS) solution and placed on a shaker (100 r/min) at 37°C for 24 h. In addition, to evaluate internal cell adhesion, scaffolds immersed in PBS with 1 μg/cm^[2]^ Fibronectin (FBN; Sciencell, USA) were set as the positive controls. First, fibronectin (FBN) was diluted with PBS solution to acquire the desirable coating solution. Second, after placing the SIS, PLLA and PLLA/Gelatin scaffolds (1.5 cm × 1.5 cm) into a 24-well plate, respectively, 1 mL of coating solution was added to each well to prepare the FBN-coated scaffold. After 24 h, all scaffolds were washed with PBS twice and placed on a new 24-well plate for cell seeding.

### Cell seeding and attachment

First, the optimal seeding density was determined according to the attachment rate of different cell densities on SIS, PLLA and PLLA/gelatin scaffolds. After cell harvesting and counting, the ASCs were resuspended in culture medium, and 1 mL of cell suspension (0.10 × 10^6^, 0.15 × 10^6^, 0.30 × 10^6^, and 0.40 × 10^6^ cells/mL) was added to the scaffold wells. The scaffolds were then placed in an incubator for culture. After 12 h, to eliminate the effect of unattached cells in the plate, all scaffolds were washed with PBS twice and placed in a new 12-well plate to determine the optimal cell density. Second, to investigate the cell adhesion property of the scaffolds, equal seeding densities of ASCs (0.15 × 10^6^ cells/mL) were added to coated and uncoated scaffolds as well as the culture dish. A cell counting kit-8 (CCK-8; Yeasen, China) was used to estimate the cell number based on the supernatant’s optical density (OD), which was read using a microplate reader (Thermo Fisher, USA) at a wavelength of 450 nm. The scaffold’s cell attachment was measured as the ratio of the OD of the scaffold to the OD of the culture dish. We also seeded cells onto scaffolds at a density of 0.15 × 10^6^ cells/mL to evaluate the effect of the coating on cell proliferation by measuring the OD on Day 2, 5, 7, and 14. The OD value of each group was normalized to Day 1 for plotting and statistics.

### Flow cytometry analysis

The phenotype maintenance of ASCs cultured on the scaffold after 14 days was analyzed by flow cytometry. The monoclonal antibodies CD44-PE, CD90-PB450, and CD105- Voilet605 (all from Biolegend, USA) were used for analyses. Cells attached to the scaffold can be easily harvested by digesting cells from the scaffold with 2.5 g/L trypsin for 3–5 min. After centrifugation, cells were resuspended using PBS with monoclonal antibodies and incubated in the dark at room temperature for 20 min. Next, cells were washed and resuspended for testing. Control samples without staining were included to confirm specificity and for compensation settings. At least 10 000 events were acquired on the flow cytometer (CytoFLEX, Beckman Coulter, USA). Data were analyzed using Flowjo V10.8.1.

### Cell proliferation and morphology

ASCs were seeded on each scaffold at a density of 0.15 × 10^6^ cells/mL per scaffold, and then the ASC-laden scaffolds were cultured *in vitro* for 1, 3, and 5 days to evaluate the cell morphology and cell adhesion area. The fluorescein isothiocyanate (FITC)-phalloidin and 4ʹ,6-diamidino-2-phenylindole dihydrochloride (DAPI) (Servicebio, China) were used to mark the cytoskeleton and nucleus of ASCs after fixing the cell-laden scaffolds overnight at 4°C with 4% (volume fraction) paraformaldehyde (Leagene, China). Additionally, ASCs were cultured for 5 days on scaffolds and labeled with CM-*DiI* (Servicebio, China) to assess cell attachment on the scaffolds. Finally, the fluorescence intensity of ASCs-scaffold complexes was visualized under *the live imager* (IVIS Lumina II).

### Evaluation of ASCs secretion and scratch assay

ASCs were cultured in the hypoxia incubator (Thermo Scientific, MA, United States) with hypoxia condition (1% O_2_), and the culture supernatants of the cells (1 × 10^6^) were collected after 1, 3, and 5 days. Subsequently, samples were centrifuged at 1500 rpm for 10 min at 4 to remove cell debris, followed by quantification of vascular endothelial growth factor (VEGF, CLOUD-CLONE CORP, Wuhan), hepatocyte growth factor (HGF, CLOUD-CLONE CORP, Wuhan) and interleukin-10 (IL-10, CLOUD-CLONE CORP, Wuhan) concentrations according to the instruction of ELISA kits. In addition, to assess the effect of ASCs on bladder epithelial cell migration, an in vitro scratch test was performed. Bladder epithelial cells were seeded into a 6-well plate at a concentration of 1 × 105 cells per well. After 24 h of culture, a straight line was drawn through the middle of the well with 200-μL pipet tips, and cells were washed thrice with PBS. Then, the 100 μL of ASCs medium supernatant was added into the bladder epithelial cell medium, and the scratch area was photographed by the inverted phase-contrast microscope at 0, 12, and 24 h.

### Scanning electron microscopy (SEM)

The microstructure analysis of the scaffolds was performed using a scanning electron microscope (SEM) (Gemini300, Zeiss, Germany). In addition, the morphology of the ASCs seeded on the scaffolds was investigated after 1, 3, and 5 days of culture to assess cell proliferation and distribution. The cell-laden scaffolds were fixed overnight at 4°C with a 2% (volume fraction) paraformaldehyde and 2.5% (volume fraction) glutaraldehyde mixture (Leagene, China), and dehydrated by soaking the samples in gradient ethanol solutions (70%, 80%, 90%, 95%, and 100%) for 10 min. Then the samples were freeze-dried and treated with spray-gold before being photographed.

### Histocompatibility

The histocompatibility and degradation of those tested materials in vivo were evaluated using subcutaneous implantation. An incision was made on the back, and ASCs-free scaffolds and ASCs-laden scaffolds (1 cm ×1 cm) were implanted subcutaneously. Then the implants and adjoining tissues were collected for histological evaluation after 6 weeks.

### Bladder augmentation model

To construct the bladder augmentation model with an ASC-free scaffold, 35 male New Zealand rabbits (2.0 ± 0.3) kg) were divided into four groups: the sham group (*n* = 5); the SIS group (*n* = 10); the PLLA group (*n* = 10); and the PLLA/Gelatin group (*n* = 10). Animals in those groups were sacrificed 6 and 12 weeks after implantation (*n* = 5 rabbits per time point). Similarly, ASCs were seeded at 0.15 × 10^6^ cells/mL and cultured for 3-5 days for the *in vivo* experiments, and then 15 male rabbits were divided into 3 groups to construct the bladder augmentation model with an ASCs-laden scaffold: ASCs-SIS group (*n* = 5); ASCs-PLLA group (*n* = 5); and ASCs-PLLA/Gelatin group (*n* = 5). Briefly, all rabbits were anesthetized with a marginal ear vein injection of α-chloralose (80 mg/kg). The urodynamic measurement was taken in each rabbit before surgery, and a median incision of the lower abdomen was made to expose the intact bladder. A longitudinal full-thickness incision (6 cm) was made in the anterior bladder wall, which was removed with silk marking stitches to create a 3 cm ×3 cm defect. Sterilized SIS, PLLA, and PLLA/Gelatin scaffolds, with or without ASCs (2 ml, 0.15 × 10^6^ cells/mL), were sutured to the defect with degradable sutures (5-0 Monocryl, Johnson & Johnson, USA). Perivesical fat was secured over the graft, and the abdomen was closed layer by layer. Penicillin (400 000 U by muscle injection, Harbin Pharmaceutical Group Holding Co., Ltd., Harbin, China) was administered daily for 3 days. All rabbits were housed individually and were sacrificed 12 weeks after implantation.

### Cystography and urodynamic examination

Cystography and urodynamics were performed for all rabbits after implantation. Under α-chloralose anesthesia, a 3-F double-lumen catheter was inserted into the bladder. Cystography was performed with 0.3 g/mL iopamidol (Aladdin, China) infused into the bladder until stress incontinence occurred. X-rays were obtained from each bladder, and then one tube of the catheter was attached to a urodynamic system (MP 150, Biopac, CA, USA) to monitor the intravesical pressure (*Pves*), and the other lumen was connected to a perfusion pump to infuse (6 mL/min) the bladder with warm saline. When the first leakage was detected, the *Pves* and maximum bladder capacity (MBC) were recorded, and bladder compliance was calculated according to the equation *∆V/∆P*, where *∆V* is volume change and is *∆P* is pressure change.

### Histologic and immunofluorescence (IF) assessment

After cystography and urodynamic analyses, the rabbits were sacrificed, and the whole bladder was obtained immediately. Bladder tissue samples were rinsed with cold saline, and tissue samples were observed macroscopically. A full-thickness sample from the transplanted site was fixed with 10% (volume fraction) formalin, dehydrated in an alcohol gradient, embedded in paraffin, and sectioned at 3-5 μm for hematoxylin and eosin (H/E) staining and Masson’s trichrome staining (MTS). Bladder regeneration was evaluated using immunofluorescence (IF) analysis with different antibodies against the urothelium (pan-cytokeratin antibody (AE1/AE3), Abcam, ab9377), smooth muscle (α-smooth muscle actin (α-SMA), Proteintech, 14395-1-AP), and vascular endothelium (cluster of differentiation 31 (CD31), Abcam, ab199012), and all sections were incubated with the secondary antibodies and DAPI. The area of positive staining and the number of vessels were quantified using Image-Pro Plus 6.0 software (Media Cybernetics, MD, USA) in ten different fields per sample.

### Statistical analysis

All data were presented as mean ± standard deviation (SD). Statistical analysis was performed using GraphPad Prism v7.0 Software. Differences between groups were analyzed using Student’s t-test or analysis of variance (ANOVA) analysis. *P*<0.05 was considered significant.

## Results

### Cytocompatibility of scaffold

**Figure 1.**
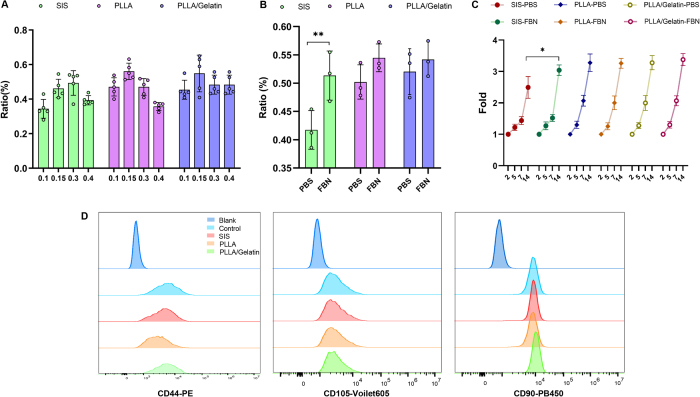
Cytocompatibility of scaffold. (A) Effect of cell number on cell attachment in uncoated scaffolds, ratio = scaffold optical density (OD) value/culture dish OD value. (B) Effect of fibronectin (FBN)-coating on cell attachment, ratio = scaffold OD value/culture dish OD value. (C) Adipose-derived stem cells (0.15 × 10^6^ cells/mL) seeded on both uncoated and FBN-coated scaffolds to study cell proliferation on Day 14 of culture. Data (a–c) are expressed as mean ± standard deviation (SD), *n* = 3. (D) Key phenotype maintenance of ASCs cultured on scaffolds for 14 d. PLLA: poly(l-lactic) acid; PBS: phosphate-buffered saline.

To determine the proper cell seeding density, we conducted cell seeding with different density suspensions. The result revealed that the cell-attachment ratio waned rapidly in the ASCs-laden scaffold at a seeding density of 0.40 × 10^6^ cells/ mL, and 0.15 × 10^6^ cell group exhibited the highest cell attachment ratio (Fig. 1A; excessive cell density reduces cell attachment). Additionally, we detected the cell attachment properties of those scaffolds by comparing the FBN-coated and FBN-uncoated scaffolds. There was no appreciable difference in cell attachment ratio between FBN-coated scaffolds and uncoated scaffolds at concentrations of 0.15 × 10^6^ cells/mL ASCs (Fig. 1B), except for the SIS scaffold. In addition, long-term culture of cells on FBN-coated and FBN-uncoated scaffolds were used to assess the effect of coating on ASCs proliferation (0.15 × 10^6^ cells/mL). However, a distinction existed solely between FBN-coated SIS and FBN-uncoated SIS, the FBN-coated PLLA and PLLA/Gelatin had no obvious differences in cell proliferation to the FBN-uncoated group, and cells from all groups were significantly expanded (Fig. 1C). Therefore, the uncoated scaffold and cell density of 0.15 × 10^6^ cells/mL were applied for the *in vivo* experiment. After 14 days of culture, ASCs from all groups still maintained high expressions of CD44, CD90, and CD105 (Fig. 1D). These results demonstrate that SIS, PLLA, and PLLA/Gelatin exhibited effective performance in ASCs attachment and proliferation, hence validating the feasibility of these scaffolds as cell-laden implantations for regeneration.

### Structure of ASCs-scaffold complex

**Figure 2.**
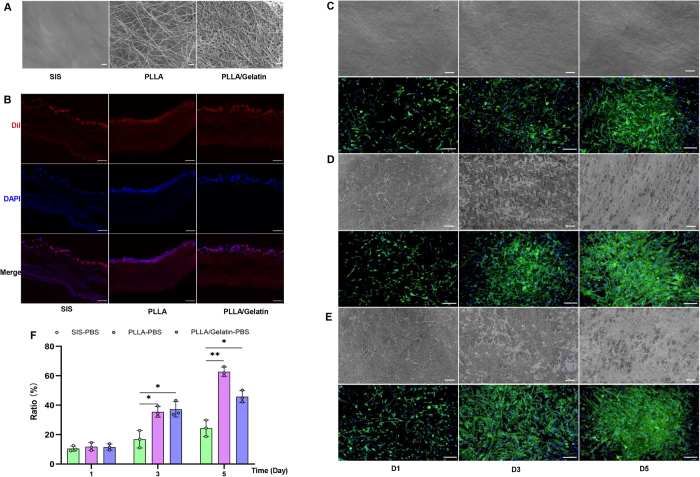
Structural properties and cell distribution on scaffold. (A) Scanning electron microscopy (SEM) micrographs of the small intestinal submucosa (SIS), poly(l-lactic) acid (PLLA), and PLLA/gelatin scaffolds. Scale bars = 10 μm. (B) DiI-labeled adipose-derived stem cells (ASCs) attach to biomaterial scaffolds. (C‒E) Distribution of ASCs on scaffolds in SEM, and cytoskeleton (green) and nucleus (blue) of ASCs attached on scaffold surface. Scale bars = 100 μm. (F) Semiquantitative evaluation of cell-spreading area based on fluorescence images. ^*^P < 0.05.

Scanning electron microscopy (SEM) images revealed that the surface of the PLLA and PLLA/Gelatin scaffolds appeared as nonwoven webs with open, interconnected pores to construct the unique 3D structure with nanofibers (Fig. 2A). Compared to the PLLA scaffold, the PLLA/Gelatin scaffold exhibited a more compact structure and reduced pore size. In contrast, the morphological properties of the SIS scaffold were distinctly different from those of the other two electrospinning scaffolds, exhibiting a flat and smooth devoid of porosity (Fig. 2A). The ASCs-laden scaffolds were cultured *in vitro*, and the microscopic examination revealed that DiI-labeled ASCs were found attached to the surface of biomaterial scaffolds (Fig. 2B). Notably, ASCs were uniformly distributed on the surface of PLLA scaffold than others. There was no fluorescence expression in the ASCs-free scaffold groups under a live imaging. Red fluorescence, originating from the *DiI*-labeled ASCs loaded in the scaffold, was observed in the scaffold materials seeded with ASCs (Supplementary Digital Content Figure S1, available at: http://links.lww.com/JS9/E820).

### Distribution of cell-laden scaffold

The normal proliferation of ASCs in scaffolds was observed on days 1, 3, and 5 using SEM and fluorescence microscopy, and cell morphology and adhesion area were also captured during *in vitro* culture (Fig. 2C-E). The figures indicated that most ASCs exhibited polygon-shape morphology and adhered to PLLA and PLLA/Gelatin nanofiber scaffolds surfaces, occupying the pore spaces and creating a cell-coated bio-surface. However, the ASCs attached to the SIS scaffold were spindle-shaped. No significant differences were observed in the number of adherent cells among the SIS, PLLA, and PLLA/Gelatin groups when ASCs were seeded on three kinds of scaffolds at the density of 0.15 × 10^6^ cells/m. Furthermore, we quantified the cells growth area to evaluate the effect of the three scaffolds on cell proliferation (Fig. 2F). Although the fluorescence area in the PLLA group increased on day 5 compared to the PLLA/gelatin group, the difference between groups was not statistically significant, and ASCs in all groups exhibited a homogenous distribution on the scaffold surface. Whereas cell fluorescence area of SIS is significantly lower than those of PLLA and PLLA/Gelatin scaffolds on days 3 and 5. Combined with the cell proliferation and ASCs flow cytometry results, SIS, PLLA and PLLA/Gelatin scaffolds can all support ASCs culture without the FBN coating, and PLLA and PLLA/Gelatin could facilitate cell proliferation and adhesion.

### Cell secretion and migration

The levels of cytokine secretion were assessed using enzyme-linked immunosorbent assays on supernatants of the hypoxia-preconditioned ASCs. Supplementary Digital Content Figure S2, available at: http://links.lww.com/JS9/E820 depicts the levels of VEGF secreted by ASCs on days 1, 3 and 5, which indicated a gradually increasing trend, and HGF and IL-10 secretion levels also exhibited a similar trend. The migration of epithelial cells was detected by a scratch assay, and the result showed that ASCs could enhance bladder epithelial cell migration compared with the PBS group (Supplementary Digital Content Figure S3A, available at: http://links.lww.com/JS9/E820), and the significant statistical difference was found between two groups (Supplementary Digital Content Figure S3B, available at: http://links.lww.com/JS9/E820).

### Histocompatibility

The histologic staining performed 6 weeks after subcutaneous SIS, PLLA, and PLLA/gelatin scaffold implantation revealed that the scaffolds attracted infiltration of inflammatory cells at their peripheries, with varying degrees of deterioration observed among the three scaffold types. Collagen fibers were deposited at the degradation site of the SIS scaffold; however, many inflammatory cells were observed around the PLLA and PLLA/Gelatin scaffolds. Conversely, three types of ASCs-laden scaffold showed a certain degree of degradation 6 weeks after subcutaneous implantation, and no significant inflammatory reaction was observed from the histologic evaluation. Pleasingly, a substantial infiltration of smooth muscle cells was observed in three ASCs-laden scaffolds (Supplementary Digital Content Figure S4, available at: http://links.lww.com/JS9/E820).

### Cystography and urodynamic evaluation

**Figure 3.**
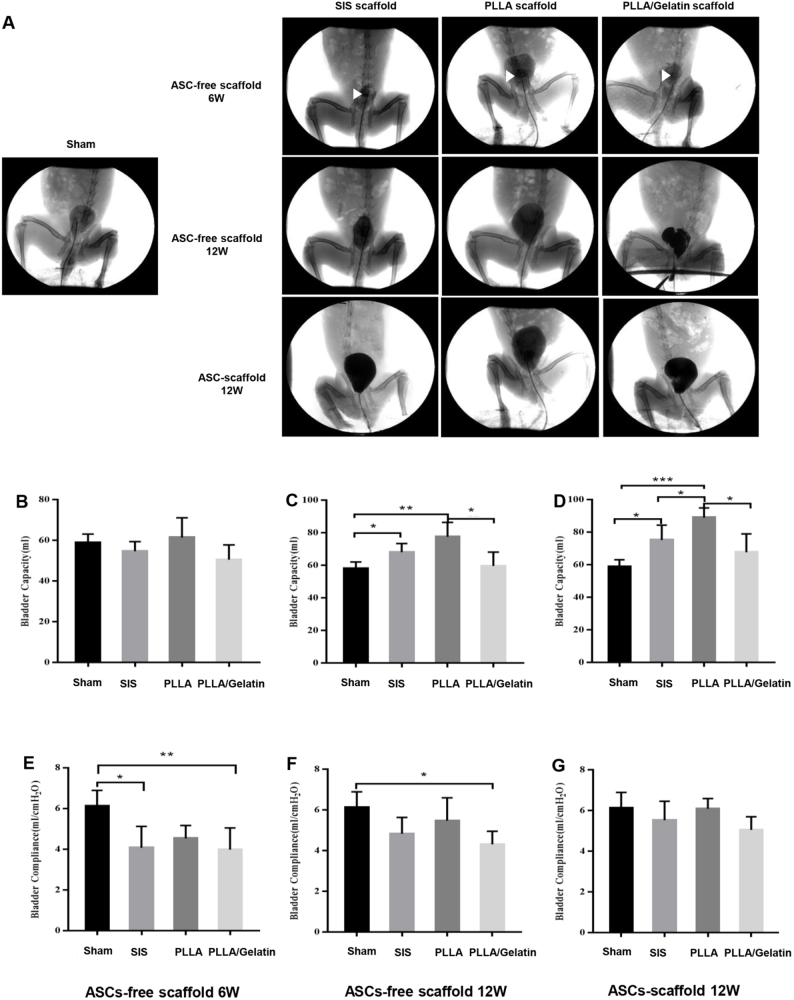
Evaluation of bladder function through cystography and urodynamics. (A) Cystography performed on different groups for evaluation of bladder morphology. 1st row: white triangle indicates bladder calculi. (B–H) The physiological function of the bladder in different groups. Data are expressed as mean ± standard deviation (SD), *n* = 5. ASC: adipose-derived stem cell; SIS: small intestinal submucosa; PLLA: poly(l-lactic) acid. ^*^P < 0.05, ^**^P < 0.01, ^***^P < 0.001.

All models underwent retrograde cystography at 6 and 12 weeks (Fig. 3A), which confirmed the absence of bladder leakage from the bladder in each group at 6 weeks. The bladder capacity of the experimental animal models constructed with the three scaffolds decreased compared with that of the sham group at 6 weeks. In addition, bladder calculi were found in the SIS, PLLA, and PLLA/Gelatin groups at 6 weeks, and the rate of bladder calculi in the three models was 40%, 20%, and 20%, respectively. An enlarger bladder with an extended, irregular profile was observed in the ASCs-free scaffold groups at 12 weeks. Despite a significant increase in the bladder capacity of the ASC-free scaffold groups at 12 weeks compared to the model groups at 6 weeks, the uneven profile of the reconstructed bladder was observed in those groups. Furthermore, reconstructed bladders in ASCs-laden scaffolds exhibited improved capacity and regular shape at 12 weeks; however, the bladder body did not completely protrude to form a smooth configuration.

The physiological function of the repaired bladder was evaluated using urodynamic tests performed at 6 and 12 weeks post-implantation. In the three categories of ASCs-free groups, there were no statistically significant differences in MBC at 6 weeks compared with the sham group (all *P>0.05*). The compliance of the regenerated bladder tissue in the three models was lower than that of the sham group (*P<0.05*; Figures 3B and E). However, MBC increased 12 weeks after reconstruction utilizing ASCs-free SIS and PLLA nanofiber scaffolds compared with the sham group ((68.04 ± 5.34) mL and (77.53 ± 8.87) mL vs. (58.88 ± 4.13) mL, respectively; *P < 0.05*). The compliance of the repaired bladder in the ASCs-free SIS and PLLA groups was similar to that of the native bladder (Fig. 3C and F). Furthermore, the MBC in the ASC-PLLA group at 12 weeks was significantly higher than that in the other groups ((88.97 ± 5.92) mL vs. (58.88 ± 4.13) mL, (75.17 ± 9.13) mL, and (61.18 ± 8.40) mL, respectively; *P < 0.05*). The compliance of the repaired bladder was similar to that of the native bladder (Fig. 3D and G). However, no significant difference was observed in bladder capacity between the ASC-PLLA/gelatin group and the sham group ((61.18 ± 8.40) mL vs. (58.88 ± 4.13) mL; *P > 0.05*).

### Macroscopy evaluation

**Figure 4.**
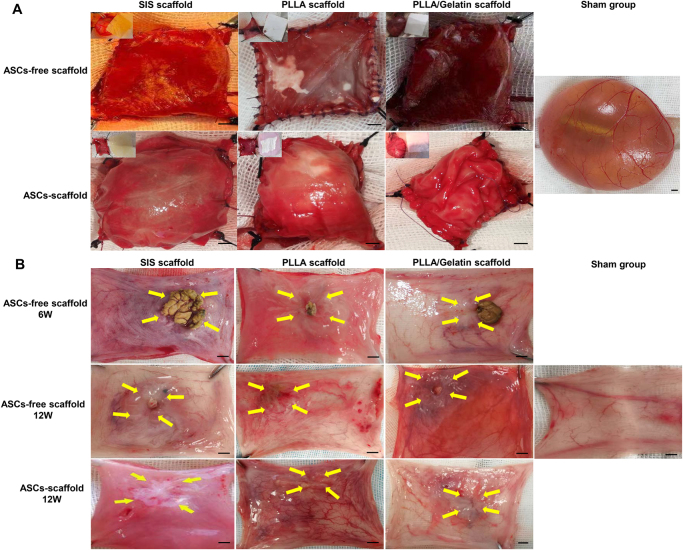
Bladder reconstruction and gross evaluation. (A) Implantation of scaffolds in rabbit bladder. (B) Gross observation of urinary bladder and stones 6 and 12 weeks postsurgery in scaffolds. Scale bar = 3 mm. ASC: adipose-derived stem cell; PLLA: poly(l-lactic) acid; SIS: small intestinal submucosa.

All bladder augmentation models were constructed by implanting three kinds of scaffolds, with or without ASCs, into the bladder (Fig. 4A). Bladder tissue from the regenerating region was harvested at predetermined intervals (ASC-free scaffold: 6 W, 12 W; ASC-laden scaffolds: 12 W). Initially, we conducted a macroscopic examination of the overall structure of the bladder. The sampling indicated that the implanted area of each group exhibited varying degrees of adhesion to peripheral tissues at all time intervals. At 6 weeks, all ASC-free scaffold groups exhibited varying degrees of contracture; however, at 12 weeks, the area of bladder contractures at the graft site diminished to some extent compared to that at 6 weeks (Fig. 4B). At 12 weeks, the scarring and shrinkage of the ASCs-laden scaffolds at the regenerated site were enhanced compared to those with ASCs-free scaffolds. Moreover, the scarring and contracture of the ASCs-PLLA group were found to be superior to those of other groups. In the study of ASCs-free scaffold transplantation, we observed bladder calculi only at 6 weeks in the SIS, PLLA nanofiber scaffold, and PLLA/Gelatin groups, and the rate of bladder calculi was approximately 40%, 20%, and 20% in the three materials, respectively.

### Histologic evaluation

**Figure 5.**
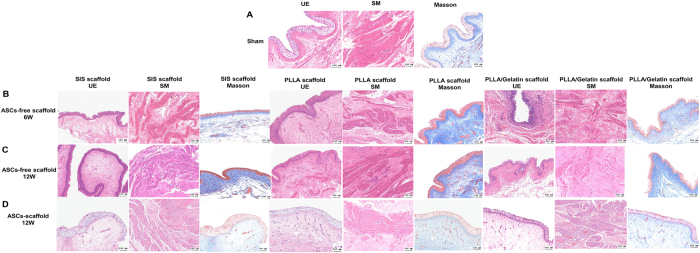
Histologic and morphologic evaluation of regenerated bladder tissue in sham and experimental groups. (A) Hematoxylin and eosin and Masson staining indicated normal bladder tissue in sham group. (B) Regenerated bladder tissue in ASC-free scaffold at 6 W. (C) Regenerated bladder tissue in ASC-free scaffold at 12 W. (D) Regenerated bladder tissue in ASC scaffold at 12 W. Scale bars = 100 μm. UE: urothelium; SM: smooth muscle; PLLA: poly(l-lactic) acid; SIS: small intestinal submucosa. ASC: adipose-derived stem cell; PLLA: poly(l-lactic) acid; SIS: small intestinal submucosa.

H/E staining revealed that the regenerated urothelium had infiltrated into the implanted regions from the native periphery tissues in all ASC-free scaffold groups at 6 weeks; nevertheless, the regenerated epithelium in each group was weak and incomplete compared with the native tissue epithelium (Fig. 5A and B). At 12 weeks, the epithelium in the regenerated regions was multilayered across all groups but lacked continuity, homogeneity, and mature differentiation for regenerated urothelium compared with the natural bladder (Fig. 5C). Disordered smooth muscle tissues and increased fibrous tissues were detected in all groups except the PLLA group in the regenerated regions. In the study of ASC-laden scaffold regeneration, the regenerated epithelium in all groups except ASC-PLLA/Gelatin resembled the native bladder epithelium (Fig. 5D). Furthermore, normal longitudinal and/or circular muscle band tissue regeneration was observed in all groups.

**Figure 6.**
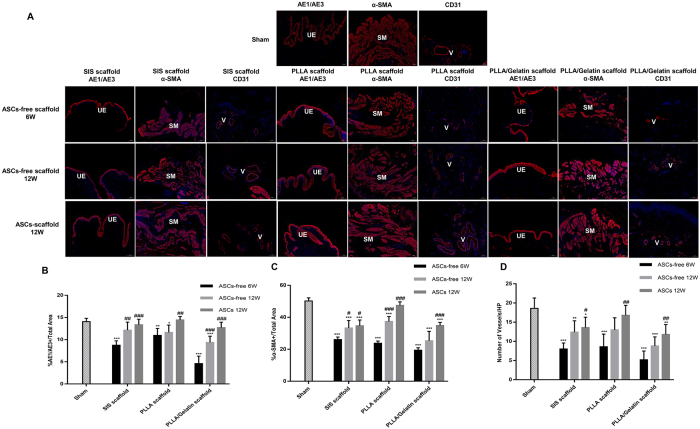
Immunofluorescence staining and quantitative assessments of regenerated bladder tissue. (A) Expression of urothelial-associated markers (AE1/AE3), smooth muscle contractile markers (α-SMA), and blood vessel endothelial marker (CD31) in regenerated bladder tissue. (B–D) Quantitative analyses of AE1/AE3 + epithelium (B), α-SMA + smooth muscle (C), and CD31 + vessel numbers (D) in regenerated bladder tissue at 6 and 12 weeks. Scale bars = 100 μm. Data are expressed as mean ± standard deviation (SD), *n* = 5. ^*^P < 0.05, ^**^P < 0.01, ^***^P < 0.001, compared with the sham group; ^#^*P* < 0.05, ^##^*P* < 0.01, ^###^*P* < 0.001, compared with ASC-free at 6 weeks. AE1/AE3: pan-cytokeratin antibody; α-SMA: α-smooth muscle actin; CD31: cluster of differentiation 31; UE: urothelium; SM: smooth muscle; V: vessels.

The bladder tissue of the regenerated area was also evaluated by IF analyses (Fig. 6A). The findings were consistent with those of H/E staining; IF staining showed that the ratios of the AE1/AE3-positive region in the three types of ASCs-free scaffolds at 12 weeks were higher than those at 6 weeks but lower than those of natural bladder tissue. The urothelial morphology of three types of ASCs-laden scaffolds was similar to that of the sham group. This result was also confirmed by quantitative morphometric analysis; the percentages of the AE1/AE3 area/total area in the three ASCs-laden groups at 12 weeks comparable to those in the sham group ((13.38 ± 1.23) %, (14.46 ± 0.76) %, and (12.73 ± 1.93)% vs. (14.06 ± 0.77)%, respectively, all *P > 0.05*), and no significant differences were observed in the three ASCs-laden groups (Fig. 6B). The ASCs-free scaffolds had also regenerated muscle fibers at 6 weeks; however, these fibers were structurally disorganized and “naive-like,” signifying incomplete regeneration.

Some disordered smooth muscle-like tissues were observed in the regenerated areas of the ASC-free groups at 12 weeks, and the α-SMA–positive smooth muscle tissues in all ASC-free groups were lower than those in the sham group (all *P < 0.05*). However, muscle staining in the ASCs-laden groups at 12 weeks indicated that the ASCs-PLLA group exhibited a smoother muscle than the ASCs-SIS and ASCs-PLLA/Gelatin groups ((47.50 ± 2.16)% vs. (34.70 ± 3.60)% and (34.86 ± 1.97%), respectively, *P* < 0.001,). Furthermore, the ratio of the α-SMA–positive area in the ASC-PLLA group was not significantly different from that of the sham group ((47.50 ± 2.16)% vs. (50.22 ± 1.98)%, *P* > 0.05) (Fig. 6C). Similarly, the density of CD31-positive vasculature progressively increased over time in SIS, PLLA, and PLLA/Gelatin groups without ASCs but remained lower than those in the sham group (all *P*<0.05). The density of newly formed capillaries in the ASC-PLLA group was significantly greater than that of the other groups, indicating that the ASC-PLLA group exhibited better neovascularization (Fig. 6A and D).

## Discussion

An appropriate barrier scaffold plays a crucial role in the tissue engineering of bladder regeneration. Currently, a diverse array of biomaterials employed in clinical practice, including SIS and PLLA-based biomimetic materials, have received significant academic interest in the field of tissue regeneration and have been demonstrated to facilitate the regeneration of different tissues^[20–23]^. However, the varying objectives and materials of the research have resulted in a deficiency of evaluative studies on the systematic safety and efficacy of constructing tissue-engineered bladders. This study systematically appraised the potential of PLLA-based biomimetic materials for bladder reconstruction compared with SIS material, aiming to select appropriate materials to guide future clinical research.

SEM demonstrated that the biodegradable nonwoven PLLA and PLLA/Gelatin nanofiber scaffold manufactured by electrospinning boasts open, highly interconnected pores and a distinctive 3D structure that resembles the ECM of the human tissue. However, the structural characteristics of the SIS scaffold were flat and smooth without a porous, dense surface. The pore size and porosity of regenerated materials are always scritical factors to consider because they provide adequate cell seeding and infiltration and increased vascular ingrowth^[24,25]^. Cell-free scaffolds (PLLA and PLLA/Gelatin) exhibited enhanced pore size and porosity, facilitating abundant ASCs to infiltrate those scaffolds, and the cell attachment rate was not significantly different from that of FBN-coated scaffolds. In addition, appropriate pore size and porosity are essential for the diffusion of nutrients, metabolic waste, and gases^[26,27]^. A previous study demonstrated that vascular smooth muscle cells aligned with synthetic microfibers 7 days after implantation^[28]^, and similar results were observed with vascular smooth muscle cells implanted on synthetic material. SEM analysis demonstrated that ASCs on PLLA fibers exhibited elongation along the longitudinal axis of the fibers. This is conducive to the spatial distribution of bladder epithelial and smooth- muscle-cell regeneration. Our data indicated that ASCs-laden scaffolds had greater bladder regeneration than other cell-free scaffolds. Furthermore, the epithelium and smooth muscle development in the models reconstructed using an ASCs-PLLA scaffold were abundant and similar to native bladder tissue compared to the ASCs-SIS and ASCs-PLLA/Gelatin groups. Of note, the regenerated bladder tissues consisting of epithelium and smooth muscle in the ASCs-PLLA/Gelatin group were inferior to those in the ASCs-SIS and ASCs-PLLA groups. We hypothesized that this may be related to the fact that the structure of PLLA/Gelatin scaffold is more compact with smaller pores. Adding gelatin to the PLLA scaffold enhances cell attachment; however, it also leads to small interfibrous pores within the PLLA/Gelatin composite, which impede cell proliferation^[29]^. In addition, the PLLA/Gelatin scaffold employed for bladder augmentation and reconstruction exhibited a certain degree of hydrophilicity, and urine infiltration in the bladder may result in the death of some ASCs on the scaffold and trigger the inflammatory reaction at the transplant site. The adverse outcome of these processes collectively hinders the effective regeneration of PLLA/gelatin scaffold.

The hydrophobic PLLA nanofiber scaffold fabricated by electrostatic spinning has hydrophobic surfaces that can serve as a barrier between fluid and viscera and prevent fluid exudation^[10,18]^. Gelatine is a hydrophilic natural macromolecule, and the surface contact angle of scaffold material can be increased by incorporating gelatin, which improves the hydrophilicity of composite material^[30]^. The gelatin-functionalized composite scaffold can not only enhance cell adhesion through improved hydrophilicity but also increase elasticity^[10]^. However, we conjecture that the PLLA/ gelatin scaffold has a degree of hydrophilicity after implantation, which may lead to urine infiltration into the graft area and the abdominal cavity under intravesical pressure conditions. This can trigger an inflammatory reaction at the graft site, compromising the efficacy of bladder tissue regeneration. Retrograde cystography also displayed an uneven overall profile, and IF analysis showed inadequate regeneration of epithelium and smooth muscle in the ASCs-PLLA/Gelatin group, potentially attributable to an exudation-related inflammatory response.

Excellent mechanical strength of tissue-engineered graft is essential for bladder tissue regeneration^[5]^. Moreover, scaffold material with favorable suture-retention and mechanical properties is also easier for surgeons to handle, which allowed the graft material to be sutured. In our study, SIS, PLLA, and PLLA/Gelatin biomimetic scaffolds exhibited satisfactory mechanical performance^[5,10,18,31]^, and no animal experienced bladder rupture. More importantly, prevention of urine leakage is crucial in bladder repair, and the appropriate physical and chemical properties of scaffold materials are essential to prevent leaks. The dense structure of SIS consisting of a four-layer tissue graft can prevent the urine traveling from the bladder to the abdominal cavity^[17]^. However, the incorporation of gelatin can cause PLLA/gelatin scaffolds to become excessively pliable and prone to tearing, thus posing challenges for suturing during surgical implantation. This may also constitute one of the underlying mechanisms contributing to suboptimal regenerative outcomes at the graft site of PLLA/gelatin scaffold.

We evaluated the effectiveness of cell-free scaffolds and cell scaffolds in bladder reconstruction. The cell-free strategy is beneficial for large–scale clinical applications, as approved scaffold products can be manufactured commercially, and surrounding cells recruited by the cell-free scaffold can achieve tissue regeneration *in vivo*. However, implanting cells into scaffold material is the most plausible and promising strategy to engineer tissues^[32,33]^. Transplant of cells cultivated on the appropriate scaffold can accelerate tissue regeneration and prevent side effects associated with cell-free scaffolds^[34]^. Native autologous cells are the optimal option due to the lack of immune rejection and ethical issues and their compatibility with the composition of bladder tissues^[35]^.

However, mature somatic cells exhibit limited capacity for *in vitro* proliferation, and their utilization is limited by extended cultivation periods^[36]^. ASCs have been widely utilized in bladder tissue regeneration if abundant and enable simple isolation and differentiation into several cell types^[34,37,38]^. One potential mechanism through which ASCs facilitate tissue regeneration is the direct differentiation of transplanted stem cells into specific cell types under the signaling cues from the surrounding bladder tissue microenvironment. Several studies have reported that stem cells can differentiate into smooth muscle and endothelial cells, facilitating the regeneration of bladder tissue^[39,40]^. However, some investigators contend that numerous beneficial effects attributed to stem cell therapy may be mediated via paracrine mechanisms, with differentiation playing a negligible role in the final regenerative effect mediated by ASCs, and only a marginal percentage of implanted stem cells survive and differentiate into smooth muscle and endothelial cells^[41]^. Importantly, ASCs exerts its therapeutic effects by secreting several molecules responsible for cell signaling, including cytokines, growth factors, chemokines, and other active substances, which have been shown to promote tissue regeneration and immunomodulation^[42]^. Our study also demonstrated that ASCs could secrete VEGF, HGF and IL-10 in hypoxic conditions, thereby facilitating cell proliferation and migration and subsequently enhancing tissue regeneration. In addition, it has been confirmed that ASCs can modulate macrophage polarization and induce the production of regulatory T cells (Treg cells) to mitigate inflammatory responses and promote tissue regeneration^[41]^. Hence, ASC transplantation is a novel method capable of regulating the inflammatory response, which may accelerate the improvement of the inflammatory microenvironment and has been confirmed to trigger migration of native cells from surrounding tissues.

Here, SIS, PLLA, and PLLA/Gelatin membranes served as the scaffold supporting the adhesion and proliferation of ASCs. The reparative effect from the morphology, histology, and function revealed that ASCs-laden scaffolds facilitated the regeneration of bladder epithelium and smooth muscle to ensure the integrity of the regenerated bladder wall. The ASCs-PLLA biomimetic scaffold, possessing a similar structure to the ECM and having similar dynamic and biodegradable properties, produced a structure similar to that of native tissue in bladder augmentation.

Bladder stones are one of the most common complications seen in models with bladder augmentation^[43]^. On one hand, rabbits are herbivores, and their urine contains high urate concentrations, predisposing them to calculi formation^[44]^; on the other hand, the presence of foreign material in the bladder can further promote bladder stones formation. Importantly, the adhesion of urate or calcium oxalate crystals to the residual biomaterial fragment in the bladder may be an essential step in the nucleation reaction leading to encrustation or stone formation^[45,46]^. Consequently, the results of our study demonstrated that vesical calculi could be detected at 6 weeks in the SIS, PLLA, and PLLA/ gelatin groups. In addition, some researchers speculate that the regeneration of the intact urothelial urothelium is closely related to the inhibition of bladder stone formation^[31]^. The results of cystography and tissue sampling have revealed that stone formation occurred in all model groups 6 weeks post-surgery. These findings were consistent with histomorphological analysis, which revealed weak and incomplete regeneration of the urothelium on the luminal side of the bladder over the transplanted graft in all groups. Bladder calculus in experimental models dissolved remarkably over time (6–12 weeks), and the regeneration of urothelium and smooth muscle in the experimental group at 12 weeks was more mature than that in the 6-week group.

In addition, the degradation rate of biomaterials is also a factor that must be considered in the formation of bladder stones. The higher percentage of calculi formation in the SIS group at 6 weeks compared to the other two groups may be attributed to the lower degradation rate of the SIS scaffold relative to that of PLLA and PLLA/Gelatin scaffolds. We hypothesized that the appropriate porosity and large surface-area-to-volume ratios may expedite the degradation of the scaffold material^[47,48]^. TEM revealed that SIS exhibited a highly smooth, dense, and homogeneous structure, with its surface/volume ratio much smaller than that of the other two groups. Finally, SIS material comprises various amino acids, including proline, glycine, and hydroxyproline, which can facilitate Ca2^+^ nucleation through electrostatic forces^[31]^. These findings are consistent with those of previous studies indicating that the incidence of calculi in porous PLLA and PLLA/Gelatin scaffolds was lower than that in the SIS group at 6 weeks.

Establishing a high-capacity, low-pressure allantoic capsule to preserve renal function is the primary objective of tissue-engineering bladder augmentation. Urodynamic evaluations are frequently used to assess changes in bladder capacity and compliance^[3]^. Our findings confirm that the bladder capacity in almost all animal models significantly increased compared with the sham group at 12 weeks; however, no significant difference was observed in bladder capacity between the reconstructed models and the sham group at 6 weeks, and bladder compliance was generally lower in the sham group. This phenomenon is consistent with the histologic evaluation, in which all experimental groups exhibited extensive regeneration of the epithelium, smooth muscle bundles, and vessels at the original defect sites at 12 weeks compared with that at 6 weeks. ASCs may facilitate the regeneration of urothelium, smooth muscle, and blood vessels^[16,49]^. These results were also inconsistent with histologic results, which revealed that the ASCs-laden models exhibited more mature urothelium and organized smooth muscle bundles, improving bladder compliance. Notably, the ASCs-PLLA group demonstrated higher bladder capacity and compliance improvement than other groups, and histomorphologic analysis revealed that the urothelium and smooth-muscle layers in the ASC-PLLA group resemble those of the sham group at the defect sites more than in the other groups.

Although our study comprehensively analyzed the utilization of SIS, PLLA, and PLLA/Gelatin in bladder augmentation and reconstruction models, several shortcomings persisted. First, we only utilized healthy animals to develop a bladder augmentation model in this study; however, the limitation was the lack of the application of the three materials in different disease backgrounds, including neurogenic bladder or bladder cancer conditions. Second, another limitation of this study is the relatively small sample size in each group, and future research will adopt a similar approach with larger sample sizes and more disease-related conditions. Also, comparative evaluations of the degradable graft materials in bladder reconstruction have been conducted, and the comparison with other biomaterials requires further study. Finally, the 12-week follow-up duration may be insufficient to evaluate the long-term durability of the intervention, and the future research should prioritize the evaluation of autologous ASCs-PLLA scaffolds with extended follow-up to ensure clinical applicability.

## Conclusion

The main objective of this work was to investigate the applicability of SIS, PLLA, and PLLA/Gelatin nanofiber scaffolds for tissue-engineered bladder augmentation and compare their efficacy in bladder reconstruction. We verified that the available PLLA nanofiber scaffold possessed appropriate mechanical properties, pore size and porosity, which could facilitate suturing, maintain bladder form, and promote the adhesion and proliferation of seeded ASCs. The analysis of ASCs-laden scaffolds demonstrated that they promote epithelium, smooth-muscle, and blood-vessel regeneration with structural and functional properties. The reconstructed tissue structure of the bladder was similar to the native bladder. In the future, the ASCs-PLLA scaffold, composed of the electrospun PLLA and autologous ASCs, is expected to be an ideal scaffold for bladder augmentation and reconstruction in clinical settings.

## Data Availability

The data are available from the corresponding author on reasonable request.

## Research registration unique identifying number (UIN)

NA.
